# Re-imagining the nation-state: An impetus from the pandemic

**DOI:** 10.3389/fsoc.2023.1086569

**Published:** 2023-02-23

**Authors:** Lorenzo Posocco, Iarfhlaith Watson

**Affiliations:** School of Sociology, University College Dublin, Dublin, Ireland

**Keywords:** nation-state, nationalism, cosmopolitanism, COVID−19, cosmopolitanism and cosmopolitics

## Abstract

In this article the positive lessons from the coronavirus pandemic are examined, focusing on the intensive activities of solidarity at the local, national, and transnational levels, the increase in scientific cooperation, the implementation of assistance policies by states, and the various endeavors of NGOs, religious communities, private organizations, wealthy and less wealthy donors, and charities to support individuals and groups affected by it. It is argued that the pandemic is not only a tragedy that revealed some of the disintegrative processes of global risk society but is also a matchless opportunity for acknowledging what can be (and is) done in the globalized world when guided by positives such as cooperation, coordination, and solidarity. Discussing the theories of globalization, nationalism, and cosmopolitanism, with special attention to Ulrich Beck's theory of reflexive society, the core point of this article is that, considering upcoming global threats of even greater magnitude, such as climate change, potentially deadlier pandemics, and nuclear conflicts, a new world order based on cooperation, coordination and solidarity between nation-states is not only desirable but necessary for survival.

## Introduction

The future cannot be a continuation of the past […] we have reached a point of historic crisis. The forces generated by the techno-scientific economy are now great enough to destroy the environment, that is to say, the material foundation of human life. The structures of human society themselves, including even some of the social foundations of the capitalist economy, are on the point of being destroyed by the erosion of what we have inherited from the human past. Our world risks both explosion and implosion. It must change (Hobsbawm, 1994, p. 584–585).

The extract from Hobsbawm brings us to the very heart of this article on the post-pandemic society, whose goal is to develop the claim that the world he described in the 1990s has not only changed, but has reached a momentum of potentially radical transformations. The starting point is the current pandemic, a more acute global threat that differs significantly from other more chronic threats, such as global warming, poverty or water deficit, which are slower and less noticeable phenomena. On the contrary, the coronavirus prompted a landscape shock (Schot and Kanger, 2018) that spread worldwide, faster than any other global threat before, bringing consequences barely imaginable in pre-pandemic society, so much so that several scientists and heads of state initially downsized its proportions, some even ridiculing those who warned against it, at least until the virus hit their countries.

The virus forced people into their homes, threatening their lives both physiologically and psychologically by turning their routines upside down, and forcing them to walk around wearing masks, something recently only seen in dystopian novels and films. At the same time, the pandemic exacerbated social inequalities with respect to housing (housing being an asset as well as a place of work and entertainment) and employment (dividing workers between essential workers and remote workers). The latter issue also magnified the problem of precarious employment. Moreover, in the period of a few weeks it brought superpowers like China, the United States of America and Europe into economic recessions, and has had an enormous impact on the global financial market, halving the price of oil worldwide, forcing transport fleets to the ground and increasing the price of basic foodstuffs such as flour and bread. Considering these effects, this article attempts to contrast evidence from a pre-pandemic world, where global risks were a matter for the “future,” with how this global shock forced all nation-states to consider global risks a priority in the “present,” and widely acknowledge that we aren't ready to deal with them.

At the same time, there is an unprecedented number of initiatives of solidarity, at the local, national and international level, within political and civil society to support those in need. These initiatives of solidarity are evidence that the twenty-first century inherited “antibodies” from previous global cataclysms such as WW2. They are manifested today in the form of intergovernmental organizations such as WHO, UN, UNICEF, but also NGOs such as Action Against Hunger, Amref, and Save the Children, whose work was crucial for coping with the effects of the pandemic. In support of this thesis, the response of civil society to the pandemic will be considered in the paper, arguing that solidarity did not come from nothing, but was based on pre-existing organizations (some that emerged post-WW2) that are changing the world for the better. Pointing to these initiatives, we will put forward the claim that an important effect of the pandemic is that it showed everybody what the world can do when highly motivated. In fact, NGOs and intergovernmental organizations weren't the only ones acting in solidarity. Numerous spontaneous transnational, bottom-up, and horizontal initiatives represent the further evidence of a world that doesn't wait for official institutions to mobilize but take the lead, motivated to help others beyond skin color and nationality.

All this doesn't translate automatically into immediate and effective positive change. In fact, if responses to the crisis reinforce—rather than change—the existing system, “its incompatibility with the natural world and its propensity to increase inequity and conflict will likely increase fragility and lead to another version of the present calamity” (Walker et al., 2020, p. 1). And yet, it would be wrong not to acknowledge that many events—including the pandemic—have increased the attention paid to global commons. Within scholarly studies, attention to global risks is a phenomenon that has existed since the 1980s and 1990s, in the works of Ulrich Beck, Craig Calhoun, Eric Hobsbawm, and others who expressed their doubts about the future of the human species. The pandemic meteorically increased this attention. Also among people at large, the role of social media has increased awareness of how interconnected and interdependent our societies are, and the necessity to take united action to prevent global disasters. Social media is at the basis of how landscape shocks such as pandemics become, to use an expression by Beck (2011), “cosmopolitan events” with a potentially explosive global reach.

The point is that the pandemic accelerated an already ongoing process of cosmopolitanization—the internalization (or embodiment) of globalization (Beck, 2011)—which in turn involves what Jurgen Habermas called “post-national consciousness” (Habermas, 2001). Post-national consciousness favors the wider acceptance that unity, solidarity, and cooperation are phenomena that go well beyond the borders of any nation-state. To paraphrase Jeffrey Alexander, the pandemic could expand the circle of the “we” (Alexander, 2016), potentially turning into an opportunity for rethinking well-established political, economic, and social models that proved inadequate to handle global threats. This would bring new original evidence supporting Beck's thesis that “the endemic nature of global risks creates a new ‘cosmopolitan civilizational shared destiny' or a new global civility” (Beck, 2011, p. 1,349).

In addition, increasing awareness urges people to exhort governments to act accordingly. This leads to the analysis of the response of politics vis-à-vis global risks. In particular, a goal in this paper is to investigate political reaction to the pandemic. The hypothesis is that some state reactions we have witnessed are a reflection of the need for a change in the direction of policies of assistance and solidarity, in clear contrast to the neoliberal economic model that has dominated the global political arena in recent times or even the liberal capitalist idea of laissez-faire. Should these experiments in social policy continue it would represent a change that, if sustained over time, could decrease the pressure of far right nationalisms whose resurgence, especially in the last two decades, must be seen through the lens of a renewed necessity of state intervention that far right parties exploited, advertising themselves as the champions of the people.

Beside looking at the reaction of politics, this paper acknowledges the existence of a body of literature supporting the thesis that the lack of international cooperation and coordination between countries is still the biggest problem hindering the development of successful solutions to global risks. This leads to the assumption that unlike civil solidarity, state solidarity is happening mostly (although not completely) within national borders, and not much at the international level, where it is left to international organizations, charities, NGOs, and wealthy (or less wealthy) private donors. Hence the necessity of rethinking the very foundations of the nation-state on the basis of a political system that must be more cooperative, coordinated, and committed to solidarity. Failing to reform the nation-state system would have a catastrophic impact, in particular vis-à-vis global warming and other ecological disasters, deadlier pandemics, but also potentially calamitous wars.^1^

Analyzing the existing literature on the issue and investigating political responses to the pandemic, it will be suggested in this paper that international coordination and cooperation are not only desirable but critical factors for avoiding bigger-scale disasters. Without such cooperation Hobsbawm's fear of world risks leading to both explosion and implosion become more likely. With this in mind, we will frame the discourse around Ulrich Beck's cosmopolitan imperative “cooperate or fail!” (Beck, 2011, p. 1,349), which the pandemic made even more urgent. Raising awareness about the priority of a radical change toward cooperation, coordination, and solidarity means acknowledging that such a step isn't just relevant for humans living in the twenty-first century but is also relevant for those in the centuries to come. In this view, the pandemic takes the shape of a modern Janus, the anthropomorphic two-faced Roman god of duality, transition, and change: an unexpected trigger for a new post-pandemic society that we'll attempt to imagine.

Finally, in the section “imagining the post-pandemic society,” the goal is to make use of the tool of sociological imagination and apply creative thinking to asking and answering questions regarding the post-pandemic society. Pointing to four key elements characterizing said society—(1) Cosmopolitan constitutionalism, (2) Cosmopolitan parties, (3) Cosmopolitan education, and (4) Methodological cosmopolitanism—the article will ask and attempt to answer these questions: Is the world moving toward the oneness of humanity, not in the sense of a centralized uniformity but of one cosmopolitan reflexive society that acts globally for the welfare of all? Wouldn't such a world be more equal, sustainable, and united? And wouldn't it be better fitted to handle global threats? How do we develop it?

## Nationalism, nation-state, globalization and the coronavirus pandemic

Originating in the Chinese region of Wuhan, the SARS-CoV-2 (severe acute respiratory syndrome coronavirus 2) resulted in the spread of the disease COVID-19 across the world, facilitated by globalization, in particular by the continuous global flow of people (and goods) that is one of its main characteristics. Over a few months, the coronavirus turned into a pandemic, bringing previously inconceivable consequences. It became an effect of what Ulrich Beck called “global risk society,” which is a society where risks—ecological, financial, military, terrorist, biochemical, and informational—are as boundless as their effects (Beck, 2012). Climate change, deforestation, water deficit, wars, toxic disposal, and pandemics are just some of the risks that borders are unable to stop and that nation-states proved, so far, to be inadequate to handle (Held, 2010).

They are inadequate because, as Conversi (2020) recently stated, national interest and divisions seem to hinder international coordination, cooperation, and solidarity, which are key elements for coping with global threats. Even a superficial look at the behavior of nation-states during the pandemic confirms his thesis. They could hardly come to an agreement about how to deal with the new virus, coordinate to limit its spread, help each other, and cooperate to find global solutions. The limits of politics in tackling global risks were well-known before the pandemic. McNeill and Engelke (2016) who defined our era as the Anthropocene—in which humans are the most powerful influence on global ecology—acknowledged several years ago that the attitudes and policies of societies toward global risks such as climate change remain doubly inconsistent, often dependent on political winds. In this view, it is not surprising that, after 6 months from the first reports of COVID-19 clusters in China, the World Health Organization (WHO) acknowledged that, although some signs of solidarity were encouraging, there have also been concerning signs of stigma, misinformation and politicization of the pandemic (WHO, 2020). Most of the events corroborating this point have been covered by the media, which broadcast speeches of powerful state leaders, like Donald Trump bickering with China, allegedly the virus super-spreader, threatening economic sanctions on those countries that shut down borders with the US, accusing WHO of being China's political marionette, and threatening to cut US funds to it.

Similar non-cooperative conduct occurred in the EU when, in the initial phases of the pandemic, the virus hit Italy, labeling the country as the virus spreader in Europe. Rather than solidarity, remarks came on 11 March 2020, from President Emmanuel Macron's spokesperson, Sibeth Ndiaye, who said that Italy didn't take the right measures that could have contained the virus. The same day, Dr. Anders Tegnell, spokesman for the Swedish Ministry of Health, flaunted the glories of Sweden stating that “the Swedish health care system is definitely much better than the Italian one in managing the contagion” (Italian Embassy in Stockholm, 2020). Italian Ambassador in Sweden, Mario Cospito, yielded to the urge to remind Dr. Tegnell that the fight against the virus is not a football game nor should EU member states chant their glories at the expense of other members, especially in a time of crisis such as the one Italy was going through. This was at a time when, in the initial phase of contagion, Italy lacked protective masks and the Italian government asked other EU members to prioritize their export to Italy. None of the EU countries answered the call, while both France and Germany temporarily blocked the exports of masks, keeping them for their national use (Repubblica, 2020). The lack of solidarity, one of the founding principles of the union, has been emphasized by its President Ursula von der Leyen, who extended a heartfelt apology to Italy, the first country hit in the EU, ‘on behalf of Europe, admitting that it had not been by its side since the beginning of the crisis' (Euronews, 2020).

In China, the dynamics around the explosion of the pandemic have raised concerns about how the lack of coordination between nation-states is detrimental to all. In particular, it is still uncertain how much time passed between the identification of the virus in China and the official warning launched by the Chinese authorities. It isn't clear why Li Wenliang, the doctor who first identified the virus and warned about its danger, was forced to sign a statement denouncing his warning as an unfounded and illegal rumor, giving more time for the spread of the virus, and why the Chinese authorities initially omitted around 50% of deaths by coronavirus (The Guardian, 2020).

Delays, oversights, and mistakes also occurred in other countries that, unlike China, weren't the first to be hit by the virus. The governments of USA, UK, Sweden, and Brazil, to mention a few noteworthy examples, came to the forefront of the media for not capitalizing on other states' experiences, scaling down the magnitude of the threat, at times ridiculing those who took strong countermeasures, advertising the positives of their national health care and belittling others'. One example among many, Prime Minister Boris Johnson stated that the UK would let the virus infect 60% of the population in order to reach herd immunity, and told the British public to prepare to “lose loved ones before their time” (CGTN, 2020). Johnson's shocking words, broadcast worldwide, did not stop countries like the USA, Sweden, and Brazil from following the same strategy. It seems fair to suggest that political failure in managing the pandemic, in particular lack of coordination, cooperation, and solidarity, had significant consequences worldwide.

In 2020, Schot et al. (2020) posited that the pandemic was functioning as a “landscape shock:” a sudden and traumatic event that affects the world at all levels, involving the social, technical and ecological environment spheres (Schot et al., 2020). This event is, among others, revealing the deep fragilities of our world of nation-states, widening cracks, and fissures of what Scambler called the “fractured society” (Scambler, 2020). Less than cooperative nation-states unprepared to deal with global risks have facilitated the spread of the virus, and intensified the landscape shock. Worse came from the weakness of those institutions that, like WHO, do not have much power to enforce countermeasures that would be beneficial for all. That power remains in the hands of nation-states: building blocks of the political world on this planet. Nation-states are sovereign, their sovereignty granted by international law, and breaking sovereignty, as seen in the ongoing Russian-Ukrainian conflict, bears consequences. Indeed, the problems of cooperation and coordination that are structural to our world of nation-states need solutions that are as urgent as they appear distant.

That being said, nation-states did not act only negatively. Beside some noteworthy cases of mismanagement, the World Health Organization reported that “many countries have implemented unprecedented measures to suppress transmission and save lives” and that “These measures have been successful in slowing the spread of the virus” (WHO, 2021). In particular, the nation-state system facilitated a quick call for national unity and solidarity that started to dominate the mass media and social media, preparing people for exceptional efforts (Malesevic, 2020). National governments used their power to close borders, shut schools and universities, ban movement, and assembly of people, which are considered by epidemiologists as fundamental countermeasures to slow down the virus (Ferguson et al., 2020). In the name of the nation, most states implemented radical measures like lockdowns and curfews, and in some cases, those who failed to comply were arrested or fined for harming public health. In many cases, exceptional funding was provided for increasing the number of intensive care beds, hiring medical personnel, buying the necessary medical equipment, building new medical facilities, and funding research to save lives. At the social and economic level, governments also enacted unprecedented assistance policies, rushing to pour money into the economy with the goal of sustaining both national business and the population at large. This is even true for unbending capitalist economies like the USA, which passed a $1 trillion stimulus proposal, half to send checks to individuals, half to backstop ailing businesses (Politico, 2020).

Similar decisions were taken by many countries in the world, suggesting that after decades of state withdrawal—a phenomenon identified, among others, by Michel Foucault and Pierre Bourdieu who wrote of “state involution” and “conservative revolution” favored by liberal capitalism (Laval, 2018)—there are signs of a renewed presence of the state in the social and economic sphere. Also at the EU level, thus at the supranational level, for the first time in history, states agreed to a common debt to tackle the crisis.

Perhaps it is too early to talk about a “pandemic revolution,” given that, for instance, a thorough look at the NextGenerationEU (the EU recovery fund) demonstrates that the EU continues to operate as a vehicle for market reforms and perceived the pandemic as an opportunity to further liberalize the market through grants and loans. However, it must be acknowledged that a EU recovery fund would have been unthinkable before, thus without, the pandemic. That said, there is enough evidence that changes are occurring, unprecedentedly, on a global scale, and that the trigger is the emergency into which the world was dragged. The pandemic put political institutions worldwide to the test, for the first time after WW2, demanding that they take action, provide answers, make projections, and give reassurance rapidly. The pandemic forced politics, in a period of a few months, to rethink well-established trends such as the capitalist idea of laissez-faire, which dominated the global political arena for decades, and to intervene strongly in the economies of states. In addition, it is questioning the privatization of health care, examining the human manipulation of nature, obliging governments to focus, more than ever, on ecological issues and potential natural disasters. It is of utmost importance that the pandemic has proven, even to traditional deniers like the USA, China, or India—which together make more than 50 percent of CO_2_ emissions in the world (Wang et al., 2019)—that global risks exist, that they are undeniably real and can potentially and fatally harm our societies. It is evident that these discourses are much more prevalent in the public debates and policies of states worldwide than they were one, ten, twenty, or 30 years ago.

In this view, the pandemic could function as a watershed in the way politics considers global risks, pressuring it to acknowledge them not as matters of a hypothetical future, but phenomena that need a solution now. As the solution to global risks lies in cooperation, coordination, and solidarity between nation-states, the pandemic could function as a force pushing politics to acknowledge the negative backdrop of existing divisions and reroute government efforts toward funding new solutions to these problems.

## The pandemic's lessons: Solidarity and cooperation at work

The social effects of the pandemic are many and interconnected. One that will have repercussions for years is the economic downturn, which already resulted in job losses and a massive increase in poverty. In the EU, Oliver Röpke—president of the Workers' Group at the European Economic and Social Committee (EESC, 2020)—stated that “If the European Central Bank's estimates are correct, the depression will mean a loss of 15% of Europe's GDP, three times the magnitude of the 2008 crisis.” “It is safe to assume that the number of jobs lost worldwide is more than 100 million” (Pizam, 2021, p. 2) Similar projections are true for other countries. Studies evidenced global losses of $600 trillion and economic growth to −6.1% (Mahapatra and Bhorekar, 2021). The global decline is the worst since the great depression.

Beside job losses and poverty, the pandemic also has other effects. Psychological stress is one of them. Fear of falling sick, losing loved ones, employment, support, and experiencing loneliness and nervousness due to social distancing and lockdown are just some of the problems. Although these effects strike all social classes without distinction, it is unquestionable that some people pay the highest price: the poor, vulnerable, children, elderly, disabled, homeless, and women (Bonaccorsi et al., 2020; Buheji et al., 2020; Van Lancker and Parolin, 2020). In many cases the pandemic worsens already problematic situations. In particular, there is special concern for the regions of the global south, where inequality is greater, people count on their daily efforts to put food on the table and cannot rely on any social safety net.

**Figure 1:**
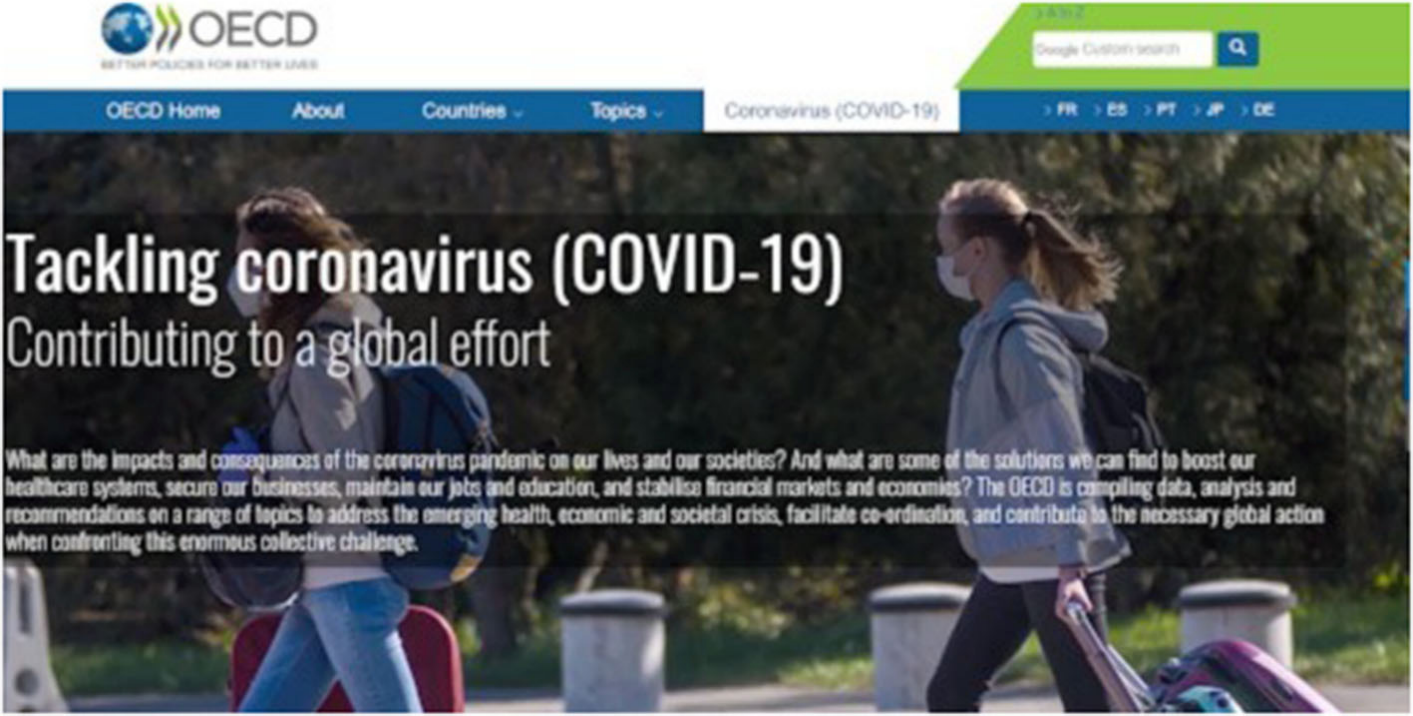
Website of the Organization for Economic Co-operation and Development (OECD), whose goal is to increase cooperation and solidarity between countries. http://oecd.org/coronavirus/en/.

**Figure 2:**
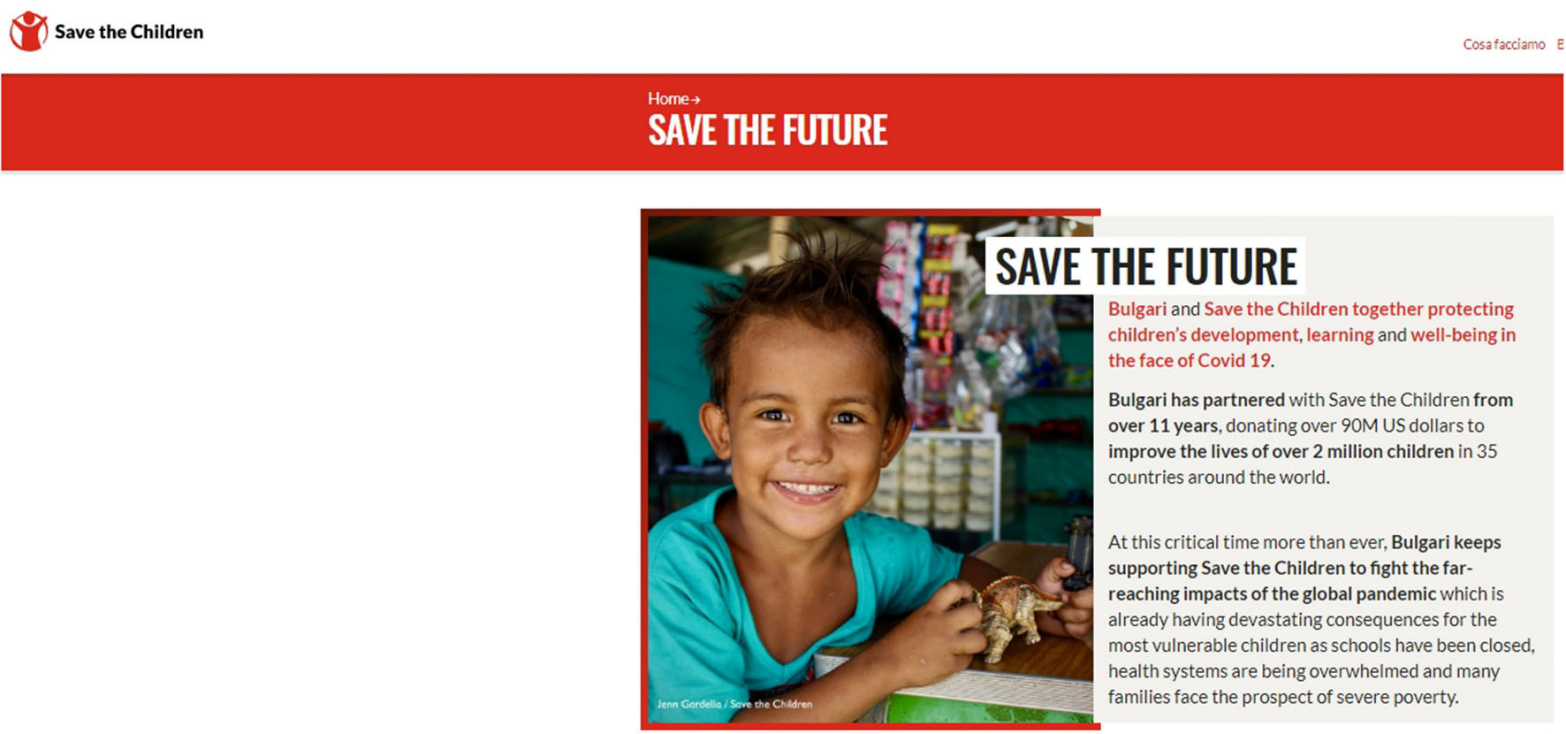
UNICEF's website addresses the fight against COVID-19 in disadvantaged areas. https://www.unicef.org/coronavirus/covid-19.

**Figure 3:**
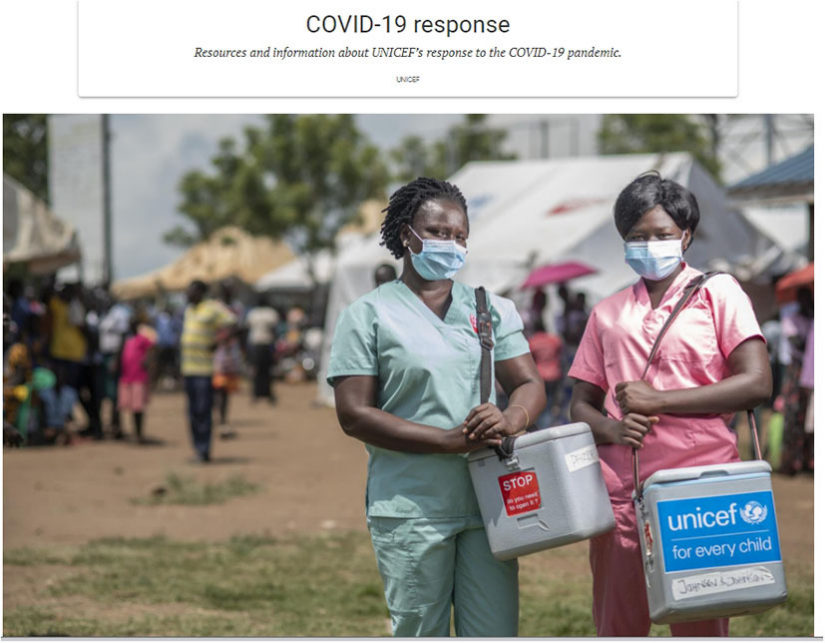
Save the Children's website calling for funding against COVID-19. https://www.savethechildren.it/save-the-future.

It is in this context of worldwide fear and concern that an unprecedented number of solidarity initiatives originated, within civil society, to help those in need. Initiatives involving religious and non-religious charities, non-governmental institutions, micro-initiatives at the family or individual level, professional associations, sport associations, foundations, social movement associations, activist groups, trade unions, etc. Non-governmental organizations such as the World Economic Forum acknowledged that incredible efforts have been made to raise unprecedented amounts of money (WEF, 2020). The same is true for the World Health Organizations, which launched the COVID-19 Solidarity Response Fund to raise money from individuals, the private sector, as well as financial and other foundations. “10 days after its March 13 launch, it had raised US$71 million from 170,000 individuals and organizations, including Facebook, Google, and FIFA” (The Lancet, 2020). It was the first time that WHO attempted to raise funding from private people, which denotes the gravity of the situation and the special measures undertaken to face a special emergency. Following WHO, UN, UNDP, OECD (see Figure 1), all the larger humanitarian organizations that were already in operation and could count on an extensive network of agencies worldwide—UNICEF (see Figure 2), Action Against Hunger, Amref, Health Communications Resources, ActionAid, Kaarvan Crafts Foundation, Phase Worldwide, Relief International, The Freedom Fund, Save the Children (see Figure 3), etc.—amplified their solidarity efforts during the pandemic. All of them started specific fundraising for tackling the consequences of the pandemic, and they did it beyond nationality, gender, language, or ethnicity. This is in line with the Habermassian idea of “postnational” or Beck's “cosmopolitanization,” potentially enlarging that “circle of we” Alexander (2012) wrote about. Indeed, the fact that the efforts of these organizations resonated through social media, journals, and televisions, increased the message of a world that needs to come together and overcome all barriers, including national and ethnic ones.

NGOs such as the Focolare Movement, a Catholic-born cross-religious, cross-cultural, and international association, is another example of an organization that prioritized the groups most affected by the pandemic, namely the poor, the “different,” and the immigrant. Founded in 1943, after the tragedies of WW2, and being present in 180 countries, it facilitated a prompt and effective action to fight the many side effects of the pandemic. Silvina Chemen, the director of Bet El, an Argentinian NGO devoted to help the poor and needy, summarized well the general sentiment among people around grassroot solidarity:

These small gestures of humanity give me hope that once the pandemic is over not only those of us who are actively engaged, but also many others, will understand how interdependent we all are. The longer we are at home alone, the more we realize we cannot do without each other [...] I renew my commitment to continue building a healed community where caring for others is our first commandment (Focolare, 2020).

Another show of solidarity that was widely broadcast by the media, but took place within the borders of a country, is the one that saw 750,000 people answering the call by the National Health System in the UK. Volunteers would undertake tasks such as delivering medication from pharmacies, driving patients to appointments, or making regular phone calls to isolated individuals (Tierney and Mahtani, 2020). According to Tierney and Mahtani's study, similar expressions of solidarity increase people's sense that they matter and their sense of participation, and have been recorded in most countries hit by the pandemic.

Also the phenomenon of private donors, wealthy philanthropists who donated to alleviate the suffering caused by the pandemic, came to the forefront of the media. The list of benefactors is long and includes, among others, celebrities such as Twitter CEO Jack Dorsey who put almost a third of his 3.6 billion dollar fortune into a fund that will tackle coronavirus relief, Bill and Melinda Gates donated $100 million through their foundation for what they defined a “once in a century pandemic,” Facebook's founder Mark Zuckerberg donated $25 million, and Ali Baba founder Jack Ma donated $14 million to develop a vaccine against the COVID-19.

**Figure 4:**
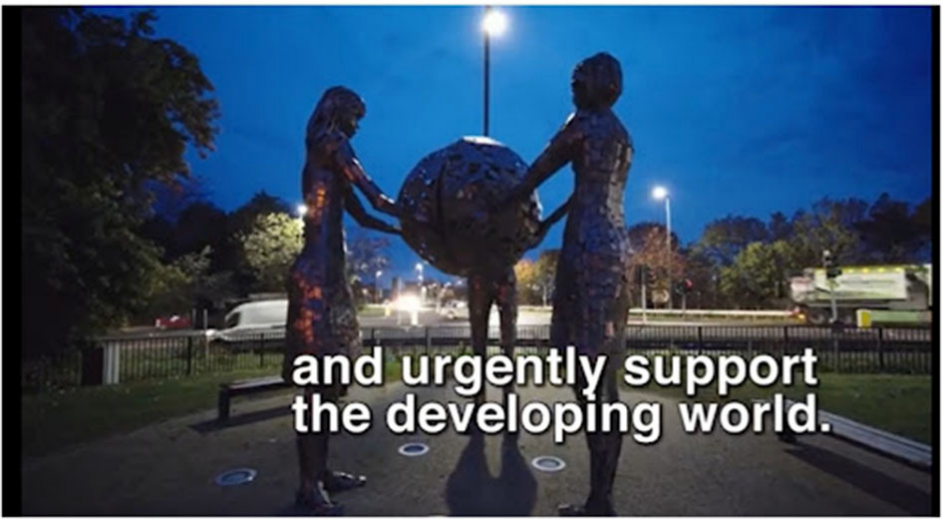
Clip 1, Interacademies call for solidarity, 2020. Click on the following link for full video https://www.youtube.com/watch?v=8loi5JECDNk.

Besides donors and NGOs, science came to the rescue with academies and research centers in search of a treatment and a vaccine against COVID-19. Scientists from all disciplines came together to tackle the consequences of the pandemic, and carry out research aimed to prevent it from happening again. The Solidarity Clinical Trial, the largest international clinical study to find an effective COVID-19 disease treatment, is one example of cooperation and coordination at the global level that is making a difference. The necessity of this study came from the lack of coordination between scientists in different countries, which led them to experiment with many individual treatments rather than join forces and come up with one valid for all. Instead, the Solidarity Clinical Trial enrolled patients in one single randomized trial that generated the strong evidence needed to determine the relative effectiveness of potential treatments (WHO Clinical Trial, 2020). A great number of medical facilities and research centers from all over the world took part in the study, and a recent investigation by Bondio and Marloth (2020) proves that this was highly beneficial, in particular by cutting the time for critical trials by 80% and providing open access data to scientists worldwide. On the wave of the Clinical Trial, many other institutions joined forces. The InterAcademy Partnership (see Figure 4) is another example, including 140 medical, scientific and engineering academies from around the world, calling on the scientific and policymaking communities to come together (Interacademies, 2020).

Similar cooperation initiatives occurred also at the micro-level, where professionals invested their time and skills to help people in need. One notable event involved a small group of engineers who, acknowledging the lack of valves for life-saving coronavirus treatment, used 3D printers to build the valves themselves, which they distributed to medical facilities (BBC, 2020).

Others focused on the environment, trying to cope with the ecological consequences of the pandemic. Although reduced transport resulted in significant reduction in air pollution and greenhouse gas emissions, the United Nations Conference on Trade and Development underlined that due to the fact that environmental protection workers were at home in lockdown, illegal deforestation, fishing and wildlife hunting increased (UNCTAD, 2020). In addition, the volume of non-recyclable waste has risen. Stay-at-home policies have increased people's consumption of take-away food delivered with single-use packaging. Also throwaway protective masks are now used daily. At a time when recycling activities have been suspended due to coronavirus, many organizations mobilized to come to the rescue of the environment.

To conclude, it seems that there's overwhelming evidence that the civil society does not wait for official institutions to mobilize, but takes the lead with the goal of lessening the suffering of fellow human beings, beyond color, gender, and nationality, and preserving life in all its forms. In this regard, and specifically in relation to the pandemic, civil society may represent a model for the political world, which is entangled in nation-centric dynamics that render the nation-state, as it is, unfit to deal successfully with, let alone prevent, global catastrophes, thus cope with the challenges of the global risk society.

## Imagining the post-pandemic world

The future of human affairs is not merely some set of variables to be predicted. The future is what is to be decided—within the limits, to be sure, of historical possibility. But this possibility is not fixed; in our time the limits seem very broad indeed (Mills, 1959, p. 174).

The goal of this section is to use the concept of “Sociological Imagination” (1959) to do what C. Wright Mills suggests in the above extract from the homonymous book. Sociological Imagination helps to theorize four elements that a post-national global society must possess to become more cooperative, internationally coordinated, equal and solidary. These elements are: (1) Cosmopolitan constitutionalism, (2) Cosmopolitan parties, (3) Cosmopolitan education, (4) Methodological cosmopolitanism.

The function of a sociological imagination is not only to render visible the invisible relationships between micro and macro phenomena, how they interact and influence each other, but also to identify what Popper (1990) called the world of propensities. The future is what is to be decided, it is open in the sense of admitting numerous possibilities that could be actualized, but the possibility of a “good future” depends, first and foremost, on the capacity of imagining it in the present. When contemplating the warning of the scientific community about the potentially catastrophic consequences of other human-led global risks such as global warming, deforestation, nuclear war, and even deadlier pandemics, the only good world is one where societies cooperate globally to avoid self-destruction. “Cooperate or fail!” wrote Ulrich Beck, who, inspired by Kant, stressed that this is the twenty-first century categorical imperative. There is no good world in the future, and there might be no world at all for human beings, without enhanced cooperation between nation-states. A critical reading of Beck suggests that, to achieve the level of cooperation required, we need to make nation-states less nationalist, to de-nationalize nation-states, and make them more cosmopolitan. We are not alone in pointing to cosmopolitanism as a viable option and attempting to imagine it. Other scholars, among whom are Calhoun (2003), Archibugi and Held (1995), and the above-mentioned Beck (2011), walked a similar path. But why cosmopolitanism?

The augmented capacity in terms of cooperation and coordination between countries, and also a different kind of political legitimacy and collective subjectivity that a cosmopolitan world involves, are powerful answers to the problems posed by nation-states entrenched in nationalism. This became even more evident in the pandemic, when, during the initial phase of contagion, a lack of cooperation and coordination between countries delayed important countermeasures that would have saved lives. In addition, priority to the nation, which is one of the main features of nationalism (Posocco and Watson, 2022), made rich nation-states race to buy their way out of the crisis before poor ones by gaining for themselves the first doses of vaccines.^2^ It isn't difficult to imagine what will happen when catastrophic events such as climate change will hit with more intensity than they already do now—when vast regions will be uninhabitable, extreme weather events will be more common, fires will destroy more forests, and droughts will jeopardize food supply (Chomsky and Pollin, 2020). Migration waves will possibly move entire populations from one region to another with consequences that, in a world dominated by national priority, can only be disastrous. Hence the need for a post-national society (Kendall et al., 2009).

In the post-national society, national identity, traditions, and values need to be reinterpreted vis-à-vis the increasingly globalized world, resulting in the intensification of worldwide social relations which link previously disparate and isolated communities on this planet and unite them into mutual dependance and unity of one world (Richter, 2017). In recent years, interregional flows of people and goods grew and reached such a speed that the local and global stopped being two distinctive and different realities. New technologies such as social media made people hyperconnected, 24–7, and any event occurring in the world can be seen anywhere exactly when it happens. Faster internet connection, better software, artificial intelligence, robotization, 3D viewers, virtual reality and other technologies gave rise to what Baldwin (2016) called telemigration, the widespread new form of existence that allows people to sit in one nation and interact (Balwin's focus was on work, but the same is true for many other activities) with people in another, or more than one nation-states at the same time. From this perspective, a larger circle of the “we” (Alexander, 2016) is already here. What is lacking is nation-state constitutions that embrace our increasingly multilingual, multiethnic, and multicultural societies and depart from the introverted nation-centric bubbles in which nationals have priority, society is divided between class A and class B citizens, the “other,” the “different,” etc. So far, nation-states have not re-modernized (Beck, 2010) enough into better versions of themselves and this has created innumerable problems, the consequences of which are very visible—one among many is the way our societies deal with immigration. Masses of migrants and refugees fleeing from hunger and poverty, children and elderly included, begging for food in rich Western societies. Others do not even make it and stop at the frontiers of states, where “walls” have been erected, protected by armed police, and others perish en route (with thousands dying in the Mediterranean sea each year).

Implementing cosmopolitan constitutions does not mean flattening cultural differences—“there can be no cosmopolitans without locals” (Hannerz, 1990, p. 239). It is fundamental to ensure that the political and juridical fields keep pace with a world that is radically changed, and will keep changing. Not to do so would maintain the status of global hysteresis (Bourdieu, 2015), a disconnect between the imagined world of nations and the reality of the global village we live in Goldin (2021). That is to say, the required shift from national exceptionalism to universalism must be a shift involving the field of law. This is very much in line with Taraborelli's reading of Kantian cosmopolitanism, where it is clear that the inclusion of “cosmopolitan right” in states' constitutions is a fundamental step in making states more cosmopolitan and less national (Taraborrelli, 2019, p. 23).

Nation-states' constitutions entrenched in nationalism legitimize and protect the status quo, hindering our increasingly multicultural, multilingual, and multi-ethnic societies from taking their place as protagonists of nation-states' constitutions. To do so, a shift toward constitutional cosmopolitanism would be instrumental. The radical change that cosmopolitan constitutions would bring is evident in the fact that they entail a legal order where the fundamental rights of every person within their jurisdiction are granted “without respect to nationality or citizenship” (Stone Sweet, 2012, p. 53). Unlike most nation-states' constitutions, cosmopolitan constitutions would ensure the Kantian emphasis on individuals as human beings rather than nationals or citizens (Kleingeld, 1998). It is a fundamental shift that doesn't require the disappearance of the “nation-state” nor the category of “national,” but it would legally empty them of their national exclusivism: a generator of inequality between nationals and non-nationals. This would have a strong impact on cultural and everyday nationalism, thus on the importance that nationals give to national tropes. Constitutional cosmopolitanism would enforce, and contribute to spreading the idea of, equality vis-à-vis the most important common denominator between all human beings: humanity.

It is not surprising that cosmopolitan constitutionalism has a bad name in law and “its tenets are routinely dismissed as naïve, sloppy, or even disingenuous” (Perju, 2013, p. 711), and why it remains relegated to the realm of “dreams,” a utopia (Kennedy, 2007). Our world is a world of nation-states driven by nationalism, and nationalism is a boundary-building phenomenon. “It locks up nation-states in themselves, making them principally worry about matters of internal security, domestic homogeneity and national growth and less about global issues and other nations' troubles” (Posocco and Watson, 2022, p. 2). Cosmopolitanism is the opposite phenomenon, it opens nation-states up, it puts national solidarity at the same level as global solidarity, and it involves a shift in terms of collective subjectivity. Cosmopolitanism assumes that nation-states and their people have obligations toward one another across, and irrespective of, national borders or nationality, while nationalism posits that nation-states have obligations, first and foremost, to the nation. It is not difficult to see how all this entails a radical change in terms of ideology that shakes the very foundations of the nation-state system as we know it, and results in a rejection of cosmopolitan constitutionalism as a utopia. Indeed, cosmopolitan constitutionalism is a necessary update to nationalism vis-à-vis the great transformations that our world went through in the last 200 years; it would provide solutions to its most problematic features. It would render nation-states less nationalist while keeping them alive and functioning.

Cosmopolitanism and its constitutionalism entail the possibility of breaking with a singular political particularity. In such a system one can be Irish, Italian, Indian or American and a “citizen of the world” at the same time, as Beck (2002, p. 19) “cosmopolitanism means: rooted cosmopolitanism, having ‘roots' and ‘wings' at the same time.” Cosmopolitanism doesn't require the rejection of one's nationality but the addition of another wider identity with potentially enormous positives for everybody. World citizenship rights would ensure every person's right and duty to participate in the authority structures and public life of any state regardless of their “historical or cultural ties to that community” (Soysal, 1994, p. 3). The introduction of such rights within cosmopolitan constitutions would have profound implications on issues of global importance such as immigration and the job market, not to mention improving a sense of belonging and solidarity that go beyond the nation. Finally, a world driven by cosmopolitan constitutionalism coupled with world citizenship rights increases the possibility of comfort and a feeling of patriotism everywhere in the world.

One of the most important challenges that cosmopolitan constitutionalism is faced with is nationalist parties. Nationalist parties are the carrier, and the most fervent advocates, of exclusivism. To use an expression by Conversi (2020), they want to fence people in, whereas to find solutions to the problems raised by nationalism we need the exact opposite. We are well-accustomed to slogans such as Make America Great Again, Make Great Britain Great Again, etc., which are at the basis of a view of the world that reinforces competitive rather than cooperative behaviors. Vis-à-vis the fact that nationalist parties are proliferating and joining forces (Jenne, 2018), and that these forces hinder a successful response to global risks, there is a need to (1) understand, and (2) challenge them. For Beck, the answer lies in internationalist parties (Beck, 2000), which should support each other on the global arena and counterbalance nationalist ones. This translates into a new deal between political parties that acknowledge the importance of international partnership as a way to enhance cooperation and decrease division around global commons.

Another important point is that we must understand how the “internalization of globalization” and the “post-national imagination” are shaped by ideological forces which favor and reproduce power relations, not cooperation. Imperialism, colonialism, and neoliberal globalization have clear forces and actors at their core that aspire to a global society with specific hierarchies in mind. For example, a recent work by Williams and Gilbert (2022) shows the dark side of the tech industry, in particular in Silicon Valley, and its connections with Wall Street. They showed how these forces helped to make the world a global village but also transformed it into one which enforces the values, pursues the interests, and maintains the worldwide position of the powerful. Numerous other works are shedding light on other forces and processes (and facilitate an understanding of what can be done about them) exacerbating divisions and hierarchies at the international levels (Davies et al., 2022; Maronitis and Pencheva, 2022; Specter, 2022).

The third element favoring cooperation and solidarity between states is cosmopolitan education. There is evidence that since the birth of the public education system in the nineteenth century, education served as a nation-building apparatus giving substance to exclusive national identity. This system is still intact and functioning, although a number of studies have shown that globalization gave rise to different forms of cosmopolitan education (Gunesh, 2004; Camicia and Zhu, 2011; Caruana, 2014; Yemini et al., 2014). Camicia and Zhu's study, in particular, investigated citizenship education in China and the USA, concluding that although nationalism remains the main discourse around which citizenship education revolves, globalization and cosmopolitanism merge within it. Students know more and more about and feel more sensitive to global commons, in particular thanks to school curricula that address these issues, although also social media play an increasingly important role (Szerszynski and Urry, 2002; Verboord, 2017; Delanty, 2018). Reforming education to further spread cosmopolitan principles would contribute to producing citizens that are more responsive to global issues and vote for parties that act accordingly. Greta Thunberg's movement “School Strike for Climate Change” is an example of how the school institution can be a force for change.

This process is definitely ongoing, but in the face of immediate global existential threats, a different pace is needed. Regarding climate change, this was expressed clearly by the sixth Intergovernmental Panel on Climate Change (IPCC) Assessment Report (IPCC, 2022). Time is over. Nation-States must act now. Governments must strengthen their globally-oriented education systems and spread cosmopolitan ideas. This step is fundamental to give birth to a post-national consciousness that is not left to chance, as it is today, but becomes part of a reflected and reflexive transition from a world of divided nation-states to a united and solidaristic one. History taught us that great harm can be done by generations raised in the principles of nationalism and its racist aberrations, Nazism and fascism among others, but we have yet to fully experience what great good could we achieve if we raise our youth in the principles of internationalism, cosmopolitanism, democracy, and global welfare.

This transition must be accompanied by a process of consciousness-raising within the social sciences too, which brings us to the fourth and last element favoring cooperation. Beck conceptualized this principle in the idea of overcoming “methodological nationalism.” Methodological nationalism is an expression used to explain the fact that social scientists assume that nation-states are the natural social and political forms of the modern world (Wimmer and Glick Schiller, 2002). As a result, their studies reflect this view, which in turn reproduces nationalism on a daily basis also in academia, which is expected to be more aware and more critical of national axioms. Delanty (2018) focused on a similar subject, highlighting the fact that narrowing social and political analysis to national horizons results in not being equipped to explain the major transformations of contemporary society. In this view, a cosmopolitan shift also in the social sciences is needed. It will help to better understand how global phenomena, including global risks, come to be, what their main properties or characteristics are, and what their significance or consequences are. Such a shift would also provide political action with the tools to fight denial and apathy, two major problems hindering successful responses to global risks, and favor transformation instead (Beck, 2011). More importantly, a better understanding of the functioning principles of global risks through lenses that are wider than the national ones will help to de-nationalize the social sciences and make them more open and responsive to cosmopolitan ideas. There are a number of problems, such as social inequality and poverty, that are mostly investigated as national issues within national borders through national lenses. This approach is problematic insofar as it frees the “national gaze [...] from looking at the misery of the world” (Beck, 2011, p. 25), and has as a consequence that the supranational logics and reasons of these phenomena remain poorly studied by scholars, who de facto legitimize them.

## Conclusions

This article suggests that the Pandemic, as a global test, is functioning as a bifurcation point and offers the opportunity to acknowledge that not only the crisis the world is facing will positively change our present, and hopefully the future, but also that, after all, not all the past is to be thrown away.

Without losing sight of the negatives, this article chose to focus on the positives stemming from the Pandemic, and acknowledged the countless initiatives of solidarity, that emerged from the political and civil world, aimed at alleviating the suffering of people, often crossing the borders of nationalism, beyond skin color, age, gender, and nationality. Most of the organizations on which this article focused, especially (but not exclusively) international NGOs, worked to alleviate the suffering of those affected the most from the Pandemic, the poor, the needy, immigrants, and all those categories already at risk. In addition, everywhere there have been initiatives not only to help other fellow human beings but also to come to the rescue of the animal world and the environment. That said, it would be wrong not to stress the good that stemmed from these people and societies coming together to withstand the shared threat posed by the pandemic. At the same time, these initiatives strengthened, and it couldn't be otherwise, many bonds of solidarity.

Focusing on long term dynamics, we suggested that many initiatives to cope with the Pandemic did not come from nothing. The organizations that support them were born in the twentieth century, in the aftermath of previous global catastrophes e.g., WW2. Societies have the capacity to learn (and they do) from events that shake up their foundations and create antibodies for the future. Given the available evidence, there is no reason to think that the Pandemic will be any different. Indeed, some evidence suggests that the Pandemic might be more than a terrible tragedy. It could be the trigger for new important changes at the systemic level bringing hope for a new and better world, in particular the return of the state as a force balancing neoliberal aspirations and a new general understanding that more cooperation and coordination between states is the recipe against the challenges that await us in the future.

At the same time, the Pandemic has shown that we are still far from solving some of the most problematic aspects of our societies. Above all, this article, which, like its authors, is strongly rooted in the tradition of nationalism studies, pointed out the problem of our system of nation-states. Facing global risks, this system, in its current form, is no longer sustainable.

And yet, we are far from having a clear answer as to how to reform it, especially in view of major global threats such as climate change. A critical reading of a recent work by Kemp et al. (2022), on the potential catastrophic scenarios emerging from climate change, suggests that it will put society to the test much more than COVID-19 did. In the context of the magnitude of such threats, humanity faces a potentially terminal cataclysm. Reforming the nation-state is an urgent necessity.

It is true, the nation-state system is not the only element hindering better responses to global threats such as pandemics or climate change. Moreover, nationalism is not the only force reproducing power relations that create inequalities and injustice, which worsen the negatives of said threats. Imperialism, colonialism and neoliberal globalization all play a role in hindering the development of better solutions and/or better mitigation strategies. There wasn't enough space in this article for a thorough analysis of the connections between nationalism and the mentioned forces. Some studies, see the recent work by HadŽidedić (2022), have begun to do it. We hope to deepen the subject in a dedicated future publication.

## Data availability statement

The original contributions presented in the study are included in the article/supplementary material, further inquiries can be directed to the corresponding author.

## Author contributions

All authors listed have made a substantial, direct, and intellectual contribution to the work and approved it for publication.

## Footnotes

^1^This article was first drafted before the ongoing Ukraine-Russian conflict, in the context of which clear references to the potential use of nuclear weapons were made. This is one manifestation of what the article was attempting to warn about.

^2^Data shows that in February 2021 over three-quarters of vaccines were available in just 10 countries that account for 60% of global GDP (World Health Organization, 2021).
